# Data library of irradiated fuel salt and off-gas tank composition for a molten salt reactor concept produced with Serpent2 and SOURCES 4C codes

**DOI:** 10.1016/j.dib.2024.110314

**Published:** 2024-03-13

**Authors:** Vaibhav Mishra, Zsolt Elter, Erik Branger, Sophie Grape, Sorouche Mirmiran

**Affiliations:** a Department of Physics and Astronomy, Applied Nuclear Physics Division, Uppsala University, Uppsala, Sweden; b Seaborg Technologies, Titangade 11 2200, Copenhagen, Denmark

**Keywords:** Nuclear safeguards, Safeguards verification, Molten salt reactors, Spent fuel, Burnup, Serpent, SOURCES 4C

## Abstract

This paper describes the methodology used to create a fuel data library comprising safeguards-relevant quantities that may be useful for verification of spent nuclear fuel (SNF) produced by simulating a concept Molten Salt Reactor (MSR). The Monte-Carlo particle transport code, Serpent2 and the calculation code SOURCES 4C were used to compile this fuel data library. The data library is based on the Compact Molten Salt Reactor (CMSR) concept being developed by Seaborg Technologies (based in Copenhagen, Denmark). The library includes data such as nuclide mass densities for a total of 1398 nuclides (in g/cm^3^), as well as total decay heat production (denoted by suffix the *‘TOT_DH’*) in Watts, total gamma photon emission rates (denoted by the suffix *‘TOT_GS’*) in photos per second, and the total activity (denoted by suffix *‘TOT_A’*) in Becquerel. Lastly, the data also includes total neutron emission rates from 1) spontaneous fission (denoted by *‘SF’* and reported in neutrons per second per cm^3^), and 2) (ɑ, n) reactions (denoted by *‘AN’* and reported in neutrons per second per cm^3^) for the fuel salt. These quantities are reported for a range of burnup-initial enrichment-cooling time (or collectively known as, BIC) parameters. The resulting fuel data library is an extension of a previously published data library for the same reactor concept but with one significant change. The current library is based on a more realistic model of the CMSR involving movement of gaseous and volatile fission products (GFP and VFP) from the core via an Off-Gas System (OGS). The dataset is made available for public use in a compressed binary format as an HDF5 (or Hierarchical Data Format) file that can be parsed using data analysis tools such as Pandas.

Specifications Table

**Table:** 

Subject	Nuclear Energy and Engineering
Specific subject area	Molten Salt Reactors, Nuclear fuel modeling, burnup studies, nuclear safeguards, volatile fission product removal.
Type of data	Table
Data format	Raw and filtered
Data collection	The dataset has been compiled using information extracted from output files (dep.m) generated by the Serpent2 calculations of fuel salt irradiation in an MSR core and `*tape 6*’ files generated by SOURCES 4C.
Data source location	Uppsala University, Uppsala, Sweden.
Data availability	Uploaded to Mendeley Data at https://data.mendeley.com/datasets/26hc75jc5j/3 DOI is 10.17632/26hc75jc5j.3

## 1 Value of the Data

• The dataset presented herein is an extension of a previously published dataset that did not account for any material movement. It is well-known that many MSR designs may opt to remove GFPs and VFPs from the fuel salt to improve the neutron economy and improve the fuel utilization since many of these GFPs and VFPs are known to have high neutron absorption cross-sections.
• The data library is similar in structure to a previously created library for the same reactor with the key differentiator that it is now created to model the operation of MSR systems with provision for online removal of gaseous effluents while the one created in [1] includes no such system. Inclusion of such a system makes the current dataset significantly larger in size compared to [1] as it has compositions of more materials than done previously.
• The data library has been developed for the CMSR concept [2] developed by Seaborg Technologies, based in Denmark. The reactor is a fluoride salt-fuelled (as FUNaK), sodium hydroxide (NaOH) moderated, thermal neutron spectrum reactor, operating on high assay-low enriched uranium (HA-LEU). The use of this library can also be extended to other similar reactors that feature removal of fission products from the primary fuel salt.
• With regards to the design, the CMSR is designed to be placed on a floating barge that enables it to be shipped to anywhere it's needed where it can anchor, connect to the grid onshore and begin generating electricity. The reactor is a novel concept, and its design offers several advantages that are unique to molten salt systems.
• The data library in question contains quantities such as isotopic mass densities, and total decay heat rates, total gamma emissions, and total activities as computed by the code, Seprent2 [3]. Furthermore, the code SOURCES 4C [4] was used with nuclide inventories from Serpent2 calculations to compute neutron emission rates from 1. The spontaneous fissions occurring in the actinides in the irradiated salt, 2. (ɑ, n) reactions occurring between alpha emissions from the major and minor actinides and the low-*Z* elements (such as fluorine) in the fuel salt matrix.
• The model used for simulation in Serpent2 also incorporates movement of GFP and VFP out of the core during irradiation via the OGS thereby making the model of the CMSR more realistic. The gaseous and volatile fission products (listed in Table 2) are removed at a range of removal rates (explained further under Data Description) and for all combinations of BIC values thereby extending the previously developed library for the CMSR which did not feature fission product removal from the salt. Lastly, the above-mentioned list of quantities are included for both, fuel salt in the primary circuit (in-core) and for the off-gas storage tank.

## 2 Background

Nuclear safeguards verification of MSR spent fuel is expected to be especially challenging in the future when these reactors enter deployment and operation. This is primarily since the spent fuel is significantly different from that produced by more conventional power operators that are in operation today. Significant efforts are needed today to further the understanding of how these reactors can be accurately modeled, what the nature of the spent fuel will be and if it can be verified using existing safeguards verification routines.

The primary objective for creation of the fuel data library presented in this paper is to enable research into the use of machine learning techniques for safeguards verification of irradiated salts. A number of such fuel libraries already exist for light water reactors (LWRs) [5,6]. There have been numerous research works looking into the possibility of using machine learning techniques for verification of spent nuclear fuel (SNF) LWRs [7], [8], [9], [10], [11], [12], [13], [14], [15] with reasonable success. However, such work looking into using conventional and possibly new verification signatures for MSRs is severely limited. Therefore, the development of this fuel library will facilitate the use of more routine signatures (based on emission from the fuel salt itself) and novel signatures (such as gamma activity of contents of the off-gas tank or calorimetry measurements of GFPs and VFPs) for safeguards verification purposes.

## 3 Data Description

The dataset is compiled as an HDF5 formatted file consisting of 1398 columns, each corresponding to the mass density of a specific isotope, labeled as either *‘fuel_isotope*’ or *‘tank_isotope*’ (each specifying the mass density of the isotope in either the fuel salt or in the off-gas tank). Thereafter, 3 columns of signatures such as the total decay heat, total gamma source rate and total activity are also included for both, the fuel salt and the off-gas tank. These columns use the following naming convention: *‘XX_TOT_YY’* where the prefix *‘XX’* specifies whether the quantity is described for the fuel salt or the off-gas tank and suffix *‘_YY’* denotes if the quantity in question is for instance, decay heat (written as *‘fuel_TOT_DH’* or *‘tank_TOT_DH’*), or gamma emission rate (written as *‘fuel_TOT_GS’* or *‘tank_TOT_GS’*) or the total activity (written as *‘fuel_TOT_A’* or *‘tank_TOT_A’*). Moreover, a select number of nuclides are removed at 35 pre-defined rates (see Table 2) of removal from the primary fuel salt to the off-gas tank and their mass densities in the tank are also recorded. The mass densities for these nuclides in the tank are recorded with the name convention *‘tank_isotope*’. Lastly, these quantities are reported at 75 burnup (BU), 41 enrichment (IE), and 8 cooling time (CT) steps and for 35 different values of GFP and VFP removal rates^1^. Therefore, in total, there are 2850 columns of data for each BIC combination with 861,000 possible combinations of BIC. The different parameters included in the dataset are further described in Table 1.

**Table 1:** Description of various columns corresponding to quantities included in the dataset.

Parameter column	Parameter description
‘BU’	Burnup (MWd/kgU)
‘IE’	Initial enrichment (wt.% U-235)
‘CT’	Cooling time (days)
‘FMI’	Flow Multiplier Index - variable that runs from 0-35 and can be used as a multiplier for computing the isotopic removal rate
‘fuel_Isotope’	1398 columns corresponding to all mass densities of isotopes tracked by Serpent2 (g/cm^3^)
‘tank_Isotope’	1398 columns corresponding to all mass densities of isotopes tracked by Serpent2 (g/cm^3^)
‘fuel_TOT_DH’	Total decay heat (Watts) produced in the fuel salt
‘fuel_TOT_GS’	Total gamma emission rate (photons/second) in the fuel salt
‘fuel_TOT_A’	Total activity (becquerel) in the fuel salt
‘tank_TOT_DH’	Total decay heat (Watts) produced in the fuel salt
‘tank_TOT_GS’	Total gamma emission rate (photons/second) in the fuel salt
‘tank_TOT_A’	Total activity (becquerel) in the fuel salt
‘flow_isotope’	Removal rates of 42 nuclides (s^−1^)
‘SF’	Total neutron emissions from all spontaneous fission reactions occurring in the irradiated primary salt
‘AN’	Total neutron emissions from (ɑ, n) reactions occurring in the irradiated primary salt

The full list of nuclides (sorted and grouped alphabetically) included in the dataset is given below:

A:

Ac-225’, ‘Ac-226’, ‘Ac-227’, ‘Ag-108’, ‘Ag-108m', ‘Ag-109’, ‘Ag-109m', ‘Ag-110’, ‘Ag-110m', ‘Ag-111’, ‘Ag-111m', ‘Ag-112’, ‘Ag-113’, ‘Ag-113m', ‘Ag-114’, ‘Ag-114m', ‘Ag-115’, ‘Ag-115m', ‘Ag-116’, ‘Ag-116m', ‘Ag-117’, ‘Ag-117m', ‘Ag-118’, ‘Ag-118m', ‘Ag-119’, ‘Ag-119m', ‘Ag-120’, ‘Ag-120m', ‘Ag-121’, ‘Ag-122’, ‘Ag-122m', ‘Ag-123’, ‘Ag-124’, ‘Ag-124m', ‘Ag-125’, ‘Ag-126’, ‘Ag-127’, ‘Ag-128’, ‘Ag-129’, ‘Ag-130’, ‘Am-241’, ‘Am-242’, ‘Am-242m', ‘Am-243’, ‘Am-244’, ‘Am-244m', ‘As-73’, ‘As-74’, ‘As-75’, ‘As-75m', ‘As-76’, ‘As-77’, ‘As-78’, ‘As-79’, ‘As-80’, ‘As-81’, ‘As-82’, ‘As-82m', ‘As-83’, ‘As-84’, ‘As-84m', ‘As-85’, ‘As-86’, ‘As-87’, ‘As-88’, ‘As-89’, ‘As-90’, ‘As-91’, ‘Au-195’, ‘Au-196’, ‘Au-196m', ‘Au-197’, ‘Au-197m', ‘Au-198’, ‘Au-198m', ‘Au-199’, ‘Au-200’, ‘Au-200m’,

B:

‘B-10’, ‘B-12’, ‘B-9’, ‘Ba-133’, ‘Ba-133m', ‘Ba-134’, ‘Ba-135’, ‘Ba-135m', ‘Ba-136’, ‘Ba-136m', ‘Ba-137’, ‘Ba-137m', ‘Ba-138’, ‘Ba-139’, ‘Ba-140’, ‘Ba-141’, ‘Ba-142’, ‘Ba-143’, ‘Ba-144’, ‘Ba-145’, ‘Ba-146’, ‘Ba-147’, ‘Ba-148’, ‘Ba-149’, ‘Ba-150’, ‘Ba-151’, ‘Ba-152’, ‘Ba-153’, ‘Be-10’, ‘Be-11’, ‘Be-12’, ‘Be-8’, ‘Be-9’, ‘Bk-249’, ‘Br-78’, ‘Br-79’, ‘Br-79m', ‘Br-80’, ‘Br-80m', ‘Br-81’, ‘Br-82’, ‘Br-82m', ‘Br-83’, ‘Br-84’, ‘Br-84m', ‘Br-85’, ‘Br-86’, ‘Br-87’, ‘Br-88’, ‘Br-89’, ‘Br-90’, ‘Br-91’, ‘Br-92’, ‘Br-93’, ‘Br-94’, ‘Br-95’, ‘Br-96’,

C:

‘C-14’, ‘C-15’, ‘C-8’, ‘Ca-50’, ‘Ca-51’, ‘Ca-52’, ‘Ca-53’, ‘Ca-54’, ‘Cd-111’, ‘Cd-111m', ‘Cd-112’, ‘Cd-113’, ‘Cd-113m', ‘Cd-114’, ‘Cd-115’, ‘Cd-115m', ‘Cd-116’, ‘Cd-117’, ‘Cd-117m', ‘Cd-118’, ‘Cd-119’, ‘Cd-119m', ‘Cd-120’, ‘Cd-121’, ‘Cd-121m', ‘Cd-122’, ‘Cd-123’, ‘Cd-123m', ‘Cd-124’, ‘Cd-125’, ‘Cd-125m', ‘Cd-126’, ‘Cd-127’, ‘Cd-128’, ‘Cd-129’, ‘Cd-129m', ‘Cd-130’, ‘Cd-131’, ‘Cd-132’, ‘Ce-138’, ‘Ce-138m', ‘Ce-139’, ‘Ce-139m', ‘Ce-140’, ‘Ce-141’, ‘Ce-142’, ‘Ce-143’, ‘Ce-144’, ‘Ce-145’, ‘Ce-146’, ‘Ce-147’, ‘Ce-148’, ‘Ce-149’, ‘Ce-150’, ‘Ce-151’, ‘Ce-152’, ‘Ce-153’, ‘Ce-154’, ‘Ce-155’, ‘Ce-156’, ‘Ce-157’, ‘Cf-249’, ‘Cf-251’, ‘Cm-240’, ‘Cm-241’, ‘Cm-242’, ‘Cm-243’, ‘Cm-244’, ‘Cm-245’, ‘Cm-246’, ‘Cm-247’, ‘Cm-248’, ‘Cm-249’, ‘Cm-250’, ‘Co-59’, ‘Co-60’, ‘Co-60m', ‘Co-61’, ‘Co-62’, ‘Co-62m', ‘Co-63’, ‘Co-64’, ‘Co-65’, ‘Co-66’, ‘Co-67’, ‘Co-68’, ‘Co-68m', ‘Co-69’, ‘Co-70’, ‘Co-70m', ‘Co-71’, ‘Co-72’, ‘Co-73’, ‘Co-74’, ‘Co-75’, ‘Cr-52’, ‘Cr-53’, ‘Cr-54’, ‘Cr-55’, ‘Cr-56’, ‘Cr-57’, ‘Cr-58’, ‘Cr-59’, ‘Cr-60’, ‘Cr-61’, ‘Cr-62’, ‘Cr-63’, ‘Cr-64’, ‘Cr-65’, ‘Cr-66’, ‘Cr-67’, ‘Cs-131’, ‘Cs-132’, ‘Cs-133’, ‘Cs-134’, ‘Cs-134m', ‘Cs-135’, ‘Cs-135m', ‘Cs-136’, ‘Cs-136m', ‘Cs-137’, ‘Cs-138’, ‘Cs-138m', ‘Cs-139’, ‘Cs-140’, ‘Cs-141’, ‘Cs-142’, ‘Cs-143’, ‘Cs-144’, ‘Cs-144m', ‘Cs-145’, ‘Cs-146’, ‘Cs-147’, ‘Cs-148’, ‘Cs-149’, ‘Cs-150’, ‘Cu-64’, ‘Cu-65’, ‘Cu-66’, ‘Cu-67’, ‘Cu-68’, ‘Cu-68m', ‘Cu-69’, ‘Cu-70’, ‘Cu-70m', ‘Cu-71’, ‘Cu-72’, ‘Cu-73’, ‘Cu-74’, ‘Cu-75’, ‘Cu-76’, ‘Cu-76m', ‘Cu-77’, ‘Cu-78’, ‘Cu-79’, ‘Cu-80’,

D:

‘Dy-159’, ‘Dy-160’, ‘Dy-161’, ‘Dy-162’, ‘Dy-163’, ‘Dy-164’, ‘Dy-165’, ‘Dy-165m', ‘Dy-166’, ‘Dy-167’, ‘Dy-168’, ‘Dy-169’, ‘Dy-170’, ‘Dy-171’, ‘Dy-172’, ‘Dy-173’,

E:

‘Er-164’, ‘Er-165’, ‘Er-166’, ‘Er-167’, ‘Er-167m', ‘Er-168’, ‘Er-169’, ‘Er-170’, ‘Er-171’, ‘Er-172’, ‘Er-173’, ‘Er-174’, ‘Er-175’, ‘Er-176’, ‘Er-177’, ‘Eu-151’, ‘Eu-152’, ‘Eu-152m', ‘Eu-153’, ‘Eu-154’, ‘Eu-154m', ‘Eu-155’, ‘Eu-156’, ‘Eu-157’, ‘Eu-158’, ‘Eu-159’, ‘Eu-160’, ‘Eu-161’, ‘Eu-162’, ‘Eu-163’, ‘Eu-164’, ‘Eu-165’, ‘Eu-166’, ‘Eu-167’,

F:

‘Fe-56’, ‘Fe-57’, ‘Fe-58’, ‘Fe-59’, ‘Fe-60’, ‘Fe-61’, ‘Fe-62’, ‘Fe-63’, ‘Fe-64’, ‘Fe-65’, ‘Fe-66’, ‘Fe-67’, ‘Fe-68’, ‘Fe-69’, ‘Fe-70’, ‘Fe-71’, ‘Fe-72’,

G:

‘Ga-68’, ‘Ga-69’, ‘Ga-70’, ‘Ga-71’, ‘Ga-72’, ‘Ga-72m', ‘Ga-73’, ‘Ga-74’, ‘Ga-74m', ‘Ga-75’, ‘Ga-76’, ‘Ga-77’, ‘Ga-78’, ‘Ga-79’, ‘Ga-80’, ‘Ga-81’, ‘Ga-82’, ‘Ga-83’, ‘Ga-84’, ‘Ga-85’, ‘Ga-86’, ‘Gd-152’, ‘Gd-154’, ‘Gd-155’, ‘Gd-155m', ‘Gd-156’, ‘Gd-157’, ‘Gd-158’, ‘Gd-159’, ‘Gd-160’, ‘Gd-161’, ‘Gd-162’, ‘Gd-163’, ‘Gd-164’, ‘Gd-165’, ‘Gd-166’, ‘Gd-167’, ‘Gd-168’, ‘Gd-169’, ‘Ge-70’, ‘Ge-71’, ‘Ge-71m', ‘Ge-72’, ‘Ge-73’, ‘Ge-73m', ‘Ge-74’, ‘Ge-75’, ‘Ge-75m', ‘Ge-76’, ‘Ge-77’, ‘Ge-77m', ‘Ge-78’, ‘Ge-79’, ‘Ge-79m', ‘Ge-80’, ‘Ge-81’, ‘Ge-81m', ‘Ge-82’, ‘Ge-83’, ‘Ge-84’, ‘Ge-85’, ‘Ge-86’, ‘Ge-87’, ‘Ge-88’, ‘Ge-89’,

H:

‘H-1’, ‘H-2’, ‘H-3’, ‘He-4’, ‘He-6’, ‘He-8’, ‘Hf-175’, ‘Hf-176’, ‘Hf-177’, ‘Hf-177m', ‘Hf-178’, ‘Hf-178m', ‘Hf-179’, ‘Hf-179m', ‘Hf-180’, ‘Hf-180m', ‘Hf-181’, ‘Hf-182’, ‘Hf-182m', ‘Hf-183’, ‘Hf-184’, ‘Hf-184m', ‘Hf-185’, ‘Hf-186’, ‘Hf-187’, ‘Hf-188’, ‘Hg-198’, ‘Hg-199’, ‘Hg-199m', ‘Hg-200’, ‘Ho-161’, ‘Ho-161m', ‘Ho-162’, ‘Ho-162m', ‘Ho-163’, ‘Ho-163m', ‘Ho-164’, ‘Ho-164m', ‘Ho-165’, ‘Ho-166’, ‘Ho-166m', ‘Ho-167’, ‘Ho-168’, ‘Ho-168m', ‘Ho-169’, ‘Ho-170’, ‘Ho-170m', ‘Ho-171’, ‘Ho-172’, ‘Ho-173’, ‘Ho-174’, ‘Ho-175’,

I:

‘I-126’, ‘I-127’, ‘I-128’, ‘I-129’, ‘I-130’, ‘I-130m', ‘I-131’, ‘I-132’, ‘I-132m', ‘I-133’, ‘I-133m', ‘I-134’, ‘I-134m', ‘I-135’, ‘I-136’, ‘I-136m', ‘I-137’, ‘I-138’, ‘I-139’, ‘I-140’, ‘I-141’, ‘I-142’, ‘I-143’, ‘I-144’, ‘In-113’, ‘In-113m', ‘In-114’, ‘In-114m', ‘In-115’, ‘In-115m', ‘In-116’, ‘In-116m', ‘In-117’, ‘In-117m', ‘In-118’, ‘In-118m', ‘In-119’, ‘In-119m', ‘In-120’, ‘In-120m', ‘In-121’, ‘In-121m', ‘In-122’, ‘In-122m', ‘In-123’, ‘In-123m', ‘In-124’, ‘In-124m', ‘In-125’, ‘In-125m', ‘In-126’, ‘In-126m', ‘In-127’, ‘In-127m', ‘In-128’, ‘In-128m', ‘In-129’, ‘In-129m', ‘In-130’, ‘In-130m', ‘In-131’, ‘In-131m', ‘In-132’, ‘In-133’, ‘In-133m', ‘In-134’, ‘In-135’, ‘Ir-189’, ‘Ir-189m', ‘Ir-190’, ‘Ir-190m', ‘Ir-191’, ‘Ir-191m', ‘Ir-192’, ‘Ir-192m', ‘Ir-193’, ‘Ir-193m', ‘Ir-194’, ‘Ir-194m', ‘Ir-195’, ‘Ir-195m', ‘Ir-196’, ‘Ir-196m', ‘Ir-197’, ‘Ir-197m',

K:

‘K-50’, ‘K-51’, ‘Kr-80’, ‘Kr-81’, ‘Kr-81m', ‘Kr-82’, ‘Kr-83’, ‘Kr-83m', ‘Kr-84’, ‘Kr-85’, ‘Kr-85m', ‘Kr-86’, ‘Kr-87’, ‘Kr-88’, ‘Kr-89’, ‘Kr-90’, ‘Kr-91’, ‘Kr-92’, ‘Kr-93’, ‘Kr-94’, ‘Kr-95’, ‘Kr-96’, ‘Kr-97’, ‘Kr-98’, ‘Kr-99’,

L:

‘La-136’, ‘La-136m', ‘La-137’, ‘La-138’, ‘La-139’, ‘La-140’, ‘La-141’, ‘La-142’, ‘La-143’, ‘La-144’, ‘La-145’, ‘La-146’, ‘La-146m', ‘La-147’, ‘La-148’, ‘La-149’, ‘La-150’, ‘La-151’, ‘La-152’, ‘La-153’, ‘La-154’, ‘La-155’, ‘Li-7’, ‘Li-8’, ‘Li-9’, ‘Lu-172’, ‘Lu-173’, ‘Lu-174’, ‘Lu-174m', ‘Lu-175’, ‘Lu-176’, ‘Lu-176m', ‘Lu-177’, ‘Lu-177m', ‘Lu-178’, ‘Lu-178m', ‘Lu-179’, ‘Lu-179m', ‘Lu-180’, ‘Lu-180m', ‘Lu-181’, ‘Lu-182’, ‘Lu-183’, ‘Lu-184’,

M:

‘Mn-54’, ‘Mn-55’, ‘Mn-56’, ‘Mn-57’, ‘Mn-58’, ‘Mn-58m', ‘Mn-59’, ‘Mn-60’, ‘Mn-60m', ‘Mn-61’, ‘Mn-62’, ‘Mn-62m', ‘Mn-63’, ‘Mn-64’, ‘Mn-65’, ‘Mn-66’, ‘Mn-67’, ‘Mn-68’, ‘Mn-69’, ‘Mo-100’, ‘Mo-101’, ‘Mo-102’, ‘Mo-103’, ‘Mo-104’, ‘Mo-105’, ‘Mo-106’, ‘Mo-107’, ‘Mo-108’, ‘Mo-109’, ‘Mo-110’, ‘Mo-111’, ‘Mo-112’, ‘Mo-113’, ‘Mo-114’, ‘Mo-115’, ‘Mo-95’, ‘Mo-96’, ‘Mo-97’, ‘Mo-98’, ‘Mo-99’,

N:

‘Nb-100’, ‘Nb-100m', ‘Nb-101’, ‘Nb-102’, ‘Nb-102m', ‘Nb-103’, ‘Nb-104’, ‘Nb-104m', ‘Nb-105’, ‘Nb-106’, ‘Nb-107’, ‘Nb-108’, ‘Nb-109’, ‘Nb-110’, ‘Nb-111’, ‘Nb-112’, ‘Nb-93’, ‘Nb-93m', ‘Nb-94’, ‘Nb-94m', ‘Nb-95’, ‘Nb-95m', ‘Nb-96’, ‘Nb-97’, ‘Nb-97m', ‘Nb-98’, ‘Nb-98m', ‘Nb-99’, ‘Nb-99m', ‘Nd-142’, ‘Nd-143’, ‘Nd-144’, ‘Nd-145’, ‘Nd-146’, ‘Nd-147’, ‘Nd-148’, ‘Nd-149’, ‘Nd-150’, ‘Nd-151’, ‘Nd-152’, ‘Nd-153’, ‘Nd-154’, ‘Nd-155’, ‘Nd-156’, ‘Nd-157’, ‘Nd-158’, ‘Nd-159’, ‘Nd-160’, ‘Nd-161’, ‘Ne-21’, ‘Ni-61’, ‘Ni-62’, ‘Ni-63’, ‘Ni-64’, ‘Ni-65’, ‘Ni-66’, ‘Ni-67’, ‘Ni-68’, ‘Ni-69’, ‘Ni-69m', ‘Ni-70’, ‘Ni-71’, ‘Ni-72’, ‘Ni-73’, ‘Ni-74’, ‘Ni-75’, ‘Ni-76’, ‘Ni-77’, ‘Ni-78’, ‘Np-235’, ‘Np-236’, ‘Np-237’, ‘Np-238’, ‘Np-239’,

O:

‘Os-186’, ‘Os-187’, ‘Os-188’, ‘Os-189’, ‘Os-189m', ‘Os-190’, ‘Os-190m’, ‘Os-191’, ‘Os-191m’, ‘Os-192’, ‘Os-192m’, ‘Os-193’, ‘Os-194’, ‘Os-195’, ‘Os-196’,

P:

‘Pa-231’, ‘Pa-232’, ‘Pa-233’, ‘Pd-105’, ‘Pd-106’, ‘Pd-107’, ‘Pd-107m', ‘Pd-108’, ‘Pd-109’, ‘Pd-109m', ‘Pd-110’, ‘Pd-111’, ‘Pd-111m', ‘Pd-112’, ‘Pd-113’, ‘Pd-113m', ‘Pd-114’, ‘Pd-115’, ‘Pd-115m', ‘Pd-116’, ‘Pd-117’, ‘Pd-117m', ‘Pd-118’, ‘Pd-119’, ‘Pd-120’, ‘Pd-121’, ‘Pd-122’, ‘Pd-123’, ‘Pd-124’, ‘Pm-146’, ‘Pm-147’, ‘Pm-148’, ‘Pm-148m', ‘Pm-149’, ‘Pm-150’, ‘Pm-151’, ‘Pm-152’, ‘Pm-152m', ‘Pm-153’, ‘Pm-154’, ‘Pm-154m', ‘Pm-155’, ‘Pm-156’, ‘Pm-157’, ‘Pm-158’, ‘Pm-159’, ‘Pm-160’, ‘Pm-161’, ‘Pm-162’, ‘Pm-163’, ‘Pr-141’, ‘Pr-142’, ‘Pr-142m', ‘Pr-143’, ‘Pr-144’, ‘Pr-144m', ‘Pr-145’, ‘Pr-146’, ‘Pr-147’, ‘Pr-148’, ‘Pr-148m', ‘Pr-149’, ‘Pr-150’, ‘Pr-151’, ‘Pr-152’, ‘Pr-153’, ‘Pr-154’, ‘Pr-155’, ‘Pr-156’, ‘Pr-157’, ‘Pr-158’, ‘Pr-159’, ‘Pt-192’, ‘Pt-193’, ‘Pt-193m', ‘Pt-194’, ‘Pt-195’, ‘Pt-195m', ‘Pt-196’, ‘Pt-197’, ‘Pt-197m', ‘Pt-198’, ‘Pt-199’, ‘Pt-199m', ‘Pu-236’, ‘Pu-237’, ‘Pu-238’, ‘Pu-239’, ‘Pu-240’, ‘Pu-241’, ‘Pu-242’, ‘Pu-243’, ‘Pu-244’, ‘Pu-246’,

R:

‘Rb-100’, ‘Rb-101’, ‘Rb-83’, ‘Rb-83m', ‘Rb-84’, ‘Rb-84m', ‘Rb-85’, ‘Rb-86’, ‘Rb-86m', ‘Rb-87’, ‘Rb-88’, ‘Rb-89’, ‘Rb-90’, ‘Rb-90m', ‘Rb-91’, ‘Rb-92’, ‘Rb-93’, ‘Rb-94’, ‘Rb-95’, ‘Rb-96’, ‘Rb-96m', ‘Rb-97’, ‘Rb-98’, ‘Rb-98m', ‘Rb-99’, ‘Re-183’, ‘Re-184’, ‘Re-184m', ‘Re-185’, ‘Re-186’, ‘Re-186m', ‘Re-187’, ‘Re-188’, ‘Re-188m', ‘Re-189’, ‘Re-190’, ‘Re-190m', ‘Re-191’, ‘Re-192’, ‘Re-193’, ‘Re-194’, ‘Rh-103’, ‘Rh-103m', ‘Rh-104’, ‘Rh-104m', ‘Rh-105’, ‘Rh-105m', ‘Rh-106’, ‘Rh-106m', ‘Rh-107’, ‘Rh-108’, ‘Rh-108m', ‘Rh-109’, ‘Rh-110’, ‘Rh-110m', ‘Rh-111’, ‘Rh-112’, ‘Rh-112m', ‘Rh-113’, ‘Rh-114’, ‘Rh-114m', ‘Rh-115’, ‘Rh-116’, ‘Rh-116m', ‘Rh-117’, ‘Rh-118’, ‘Rh-119’, ‘Rh-120’, ‘Rh-121’, ‘Rh-122’, ‘Ru-100’, ‘Ru-101’, ‘Ru-102’, ‘Ru-103’, ‘Ru-103m', ‘Ru-104’, ‘Ru-105’, ‘Ru-106’, ‘Ru-107’, ‘Ru-108’, ‘Ru-109’, ‘Ru-110’, ‘Ru-111’, ‘Ru-112’, ‘Ru-113’, ‘Ru-113m', ‘Ru-114’, ‘Ru-115’, ‘Ru-116’, ‘Ru-117’, ‘Ru-118’, ‘Ru-119’, ‘Ru-120’,

S:

‘Sb-118’, ‘Sb-118m', ‘Sb-119’, ‘Sb-119m', ‘Sb-120’, ‘Sb-120m', ‘Sb-121’, ‘Sb-122’, ‘Sb-122m', ‘Sb-123’, ‘Sb-124’, ‘Sb-124m', ‘Sb-125’, ‘Sb-126’, ‘Sb-126m', ‘Sb-127’, ‘Sb-128’, ‘Sb-128m', ‘Sb-129’, ‘Sb-129m', ‘Sb-130’, ‘Sb-130m', ‘Sb-131’, ‘Sb-132’, ‘Sb-132m', ‘Sb-133’, ‘Sb-134’, ‘Sb-134m', ‘Sb-135’, ‘Sb-136’, ‘Sb-137’, ‘Sb-138’, ‘Sb-139’, ‘Sc-50’, ‘Sc-50m', ‘Sc-51’, ‘Sc-52’, ‘Sc-53’, ‘Sc-54’, ‘Sc-55’, ‘Sc-56’, ‘Sc-57’, ‘Se-75’, ‘Se-76’, ‘Se-77’, ‘Se-77m', ‘Se-78’, ‘Se-79’, ‘Se-79m', ‘Se-80’, ‘Se-81’, ‘Se-81m', ‘Se-82’, ‘Se-83’, ‘Se-83m', ‘Se-84’, ‘Se-85’, ‘Se-86’, ‘Se-87’, ‘Se-88’, ‘Se-89’, ‘Se-90’, ‘Se-91’, ‘Se-92’, ‘Se-93’, ‘Se-94’, ‘Sm-147’, ‘Sm-148’, ‘Sm-149’, ‘Sm-150’, ‘Sm-151’, ‘Sm-152’, ‘Sm-153’, ‘Sm-153m', ‘Sm-154’, ‘Sm-155’, ‘Sm-156’, ‘Sm-157’, ‘Sm-158’, ‘Sm-159’, ‘Sm-160’, ‘Sm-161’, ‘Sm-162’, ‘Sm-163’, ‘Sm-164’, ‘Sm-165’, ‘Sn-115’, ‘Sn-116’, ‘Sn-117’, ‘Sn-117m', ‘Sn-118’, ‘Sn-119’, ‘Sn-119m', ‘Sn-120’, ‘Sn-121’, ‘Sn-121m', ‘Sn-122’, ‘Sn-123’, ‘Sn-123m', ‘Sn-124’, ‘Sn-125’, ‘Sn-125m', ‘Sn-126’, ‘Sn-127’, ‘Sn-127m', ‘Sn-128’, ‘Sn-128m', ‘Sn-129’, ‘Sn-129m', ‘Sn-130’, ‘Sn-130m', ‘Sn-131’, ‘Sn-131m', ‘Sn-132’, ‘Sn-133’, ‘Sn-134’, ‘Sn-135’, ‘Sn-136’, ‘Sn-137’, ‘Sr-100’, ‘Sr-101’, ‘Sr-102’, ‘Sr-103’, ‘Sr-104’, ‘Sr-85’, ‘Sr-85m', ‘Sr-86’, ‘Sr-87’, ‘Sr-87m', ‘Sr-88’, ‘Sr-89’, ‘Sr-90’, ‘Sr-91’, ‘Sr-92’, ‘Sr-93’, ‘Sr-94’, ‘Sr-95’, ‘Sr-96’, ‘Sr-97’, ‘Sr-98’, ‘Sr-99’,

T:

‘Ta-178’, ‘Ta-178m', ‘Ta-179’, ‘Ta-179m', ‘Ta-180’, ‘Ta-180m', ‘Ta-181’, ‘Ta-182’, ‘Ta-182m', ‘Ta-183’, ‘Ta-184’, ‘Ta-185’, ‘Ta-185m', ‘Ta-186’, ‘Ta-187’, ‘Ta-188’, ‘Ta-189’, ‘Ta-190’, ‘Tb-156’, ‘Tb-156m', ‘Tb-157’, ‘Tb-158’, ‘Tb-158m', ‘Tb-159’, ‘Tb-160’, ‘Tb-161’, ‘Tb-162’, ‘Tb-163’, ‘Tb-164’, ‘Tb-165’, ‘Tb-166’, ‘Tb-167’, ‘Tb-168’, ‘Tb-169’, ‘Tb-170’, ‘Tb-171’, ‘Tc-100’, ‘Tc-101’, ‘Tc-102’, ‘Tc-102m', ‘Tc-103’, ‘Tc-104’, ‘Tc-105’, ‘Tc-106’, ‘Tc-107’, ‘Tc-108’, ‘Tc-109’, ‘Tc-110’, ‘Tc-111’, ‘Tc-112’, ‘Tc-113’, ‘Tc-114’, ‘Tc-115’, ‘Tc-116’, ‘Tc-117’, ‘Tc-118’, ‘Tc-98’, ‘Tc-99’, ‘Tc-99m', ‘Te-120’, ‘Te-121’, ‘Te-121m', ‘Te-122’, ‘Te-123’, ‘Te-123m', ‘Te-124’, ‘Te-125’, ‘Te-125m', ‘Te-126’, ‘Te-127’, ‘Te-127m', ‘Te-128’, ‘Te-129’, ‘Te-129m', ‘Te-130’, ‘Te-131’, ‘Te-131m', ‘Te-132’, ‘Te-133’, ‘Te-133m', ‘Te-134’, ‘Te-135’, ‘Te-136’, ‘Te-137’, ‘Te-138’, ‘Te-139’, ‘Te-140’, ‘Te-141’, ‘Te-142’, ‘Th-227’, ‘Th-228’, ‘Th-229’, ‘Th-230’, ‘Th-232’, ‘Th-233’, ‘Th-234’, ‘Ti-50’, ‘Ti-51’, ‘Ti-52’, ‘Ti-53’, ‘Ti-54’, ‘Ti-55’, ‘Ti-56’, ‘Ti-57’, ‘Ti-58’, ‘Ti-59’, ‘Ti-60’, ‘Ti-61’, ‘Tm-166’, ‘Tm-167’, ‘Tm-168’, ‘Tm-169’, ‘Tm-170’, ‘Tm-171’, ‘Tm-172’, ‘Tm-173’, ‘Tm-174’, ‘Tm-175’, ‘Tm-176’, ‘Tm-177’, ‘Tm-178’, ‘Tm-179’,

U:

‘U-232’, ‘U-233’, ‘U-234’, ‘U-235’, ‘U-236’, ‘U-237’, ‘U-238’,

V:

‘V-50’, ‘V-51’, ‘V-52’, ‘V-53’, ‘V-54’, ‘V-55’, ‘V-56’, ‘V-57’, ‘V-58’, ‘V-59’, ‘V-60’, ‘V-61’, ‘V-62’, ‘V-63’, ‘V-64’, ‘V-65’,

W:

‘W-180’, ‘W-181’, ‘W-182’, ‘W-183’, ‘W-183m', ‘W-184’, ‘W-185’, ‘W-185m', ‘W-186’, ‘W-186m', ‘W-187’, ‘W-188’, ‘W-189’, ‘W-190’, ‘W-190m', ‘W-191’, ‘W-192’,

X:

‘Xe-128’, ‘Xe-129’, ‘Xe-129m', ‘Xe-130’, ‘Xe-131’, ‘Xe-131m', ‘Xe-132’, ‘Xe-132m', ‘Xe-133’, ‘Xe-133m', ‘Xe-134’, ‘Xe-134m', ‘Xe-135’, ‘Xe-135m', ‘Xe-136’, ‘Xe-137’, ‘Xe-138’, ‘Xe-139’, ‘Xe-140’, ‘Xe-141’, ‘Xe-142’, ‘Xe-143’, ‘Xe-144’, ‘Xe-145’, ‘Xe-146’, ‘Xe-147’,

Y:

‘Y-100’, ‘Y-100m', ‘Y-101’, ‘Y-102’, ‘Y-102m', ‘Y-103’, ‘Y-104’, ‘Y-105’, ‘Y-106’, ‘Y-107’, ‘Y-88’, ‘Y-88m', ‘Y-89’, ‘Y-89m', ‘Y-90’, ‘Y-90m', ‘Y-91’, ‘Y-91m', ‘Y-92’, ‘Y-93’, ‘Y-93m', ‘Y-94’, ‘Y-95’, ‘Y-96’, ‘Y-96m', ‘Y-97’, ‘Y-97m', ‘Y-98’, ‘Y-98m', ‘Y-99’, ‘Yb-169’, ‘Yb-170’, ‘Yb-171’, ‘Yb-171m', ‘Yb-172’, ‘Yb-173’, ‘Yb-174’, ‘Yb-175’, ‘Yb-175m', ‘Yb-176’, ‘Yb-176m', ‘Yb-177’, ‘Yb-177m', ‘Yb-178’, ‘Yb-179’, ‘Yb-180’, ‘Yb-181’,

Z:

‘Zn-66’, ‘Zn-67’, ‘Zn-68’, ‘Zn-69’, ‘Zn-69m', ‘Zn-70’, ‘Zn-71’, ‘Zn-71m', ‘Zn-72’, ‘Zn-73’, ‘Zn-73m', ‘Zn-74’, ‘Zn-75’, ‘Zn-76’, ‘Zn-77’, ‘Zn-77m', ‘Zn-78’, ‘Zn-79’, ‘Zn-80’, ‘Zn-81’, ‘Zn-82’, ‘Zn-83’, ‘Zr-100’, ‘Zr-101’, ‘Zr-102’, ‘Zr-103’, ‘Zr-104’, ‘Zr-105’, ‘Zr-106’, ‘Zr-107’, ‘Zr-108’, ‘Zr-109’, ‘Zr-91’, ‘Zr-92’, ‘Zr-93’, ‘Zr-94’, ‘Zr-95’, ‘Zr-96’, ‘Zr-97’, ‘Zr-98’, ‘Zr-99’.

The full list of nuclides removed from the primary salt and tracked in the off-gas tank is given below:

‘I135’, ‘I137’, ‘I138’, ‘I136’, ‘I136m', ‘I139’, ‘I134’, ‘I134m', ‘I140’, ‘I133’, ‘Kr90’, ‘Kr89’, ‘Kr91’, ‘Kr92’, ‘Kr88’, ‘Kr93’, ‘Kr87’, ‘Kr94’, ‘Kr86’, ‘Kr96’, ‘Kr85’, ‘Xe138’, ‘Xe139’, ‘Xe140’, ‘Xe137’, ‘Xe136’, ‘Xe141’, ‘Xe142’, ‘Xe135m', ‘Xe135’, 'Cs137’, 'Cs140’, ‘Nb95’, ‘Zr95’, ‘Sr91’, ‘Sr89’, ‘Tc103’, ‘Mo99’, ‘Pr147’, ‘Y95’, ‘Sb132’, ‘Sb129’

The BU values in the data library were chosen in a manner that there were a total of 75 burnup steps starting at 10^−9^ MWd/kgU (or 1 second of reactor operation) and going up to roughly 29 MWd/kgU. Of these, 25 steps correspond to values below 1 MWd/kgU which increase by a factor of roughly 2.5 after each interval. From burnup values³ 1 MWd/kgU, the values increase by roughly 0.5 MWd/kgU at each step. Similarly, the IE parameter varies between 10.0 and 20.0 wt.% U-235 in steps of 0.25 which implies that there are 41 steps covering the full range of IE in total. Furthermore, for CT, the nuclide inventories and the associated values of DH, SF, and GS for the primary salt and the off-gas nuclides were computed at a total of 8 steps. These CT values range between roughly 0 days and up to 10 years in 8 steps. Lastly, the GFP removal rates are selected in a range of 35 steps that vary from a set initial value (from Table 2) and by a factor of up to 50 at the last step. The initial values of the removal rates were selected based on trial-and-error runs using the reprocessor feature in Serpent2 and the nuclides were selected based on scoping studies conducted by Seaborg. Some of these values may be unphysically high or low and the actual values to be used in the CMSR will be decided at a later stage. These parameters are summarized in Table 2.

**Table 2:** Key dataset parameters used for setting up Serpent2 cases.

Parameter Name	Parameter Value
Burnup (B)	Range: 0.0 – 29.0 MWd/kgU 75 steps in total For BU < 1.0 MWd/kgU – steps increasing by a factor of 2.5 For BU ≥ 1.0 MWd/kgU – steps of 0.5 MWd/kgU
Initial enrichment (I)	Range: 10.0 – 20.0 wt.% U-235 (41 steps in total) Steps of 0.25 wt.% U-235
Cooling time (C)	Range: 0 – 10 years (8 steps in total) 0, 1, 30, 365, 730, 1825, 2920, and 3650 days
Flow rate multiplier (Φ) (for off-gas removal)	Range: Different for each nuclide and follows the following relation: 1 ≤ Φ ≤ 50, in 35 steps
OGS nuclides and respective initial removal rates (Φ_i_) (in sec^−1^) *‘m’ denotes metastable nuclide	I-135 8.08E-17 I-137 7.34E-17 I-138 4.10E-17 I-136 3.75E-17 I-136m 3.52E-17 I-139 2.22E-17 I-134 1.43E-17 I-134m 1.03E-17 I-140 4.46E-18 I-133 2.55E-18 Kr-90 1.19E-18 Kr-89 9.15E-19 Kr-91 8.67E-19 Kr-92 4.63E-19 Kr-88 4.61E-19 Kr-93 1.41E-19 Kr-87 1.23E-19 Kr-94 2.98E-20 Kr-86 2.43E-20 Kr-96 9.85E-21 Kr-85 6.54E-21 Xe-138 1.30E-18 Xe-139 1.18E-18 Xe-140 9.64E-19 Xe-137 8.70E-19 Xe-136 5.83E-19 Xe-141 3.50E-19 Xe-142 1.32E-19 Xe-135m 4.90E-20 Xe-135 2.22E-20 Cs-137 2.00E-20 Cs-140 5.78E-19 Nb-95 2.80E-23 Zr-95 3.26E-20 Sr-91 7.31E-20 Sr-89 4.47E-21 Tc-103 2.11E-20 Mo-99 1.09E-20 Pr-147 9.15E-18 Y-95 3.00E-19 Sb-132 3.68E-19 Sb-129 1.98E-20

The dataset can be read in using widely used software libraries used for data manipulation and analysis such as Pandas. It should be noted that HDF5 files use a binary format of storing data (which is highly compressed) and cannot be read by everyday text editors such as Microsoft Word. Once the dataset is read in, the user can then choose, for instance to look only at columns with isotopes contained in the primary salt in the following manner:

import pandas as pd

df = pd.read_hdf(*'path/to/dataset.hdf5’*)

fuel_columns = df.filter(like=’fuel_’) or

tank_columns = df.filter(like=’tank_’)

The user may then create subsets of the dataset by selecting the enrichment and cooling time (IE = 20.0 wt.% U-235 and CT = 0.0 in this case) as follows:

dfIE12CT0 = df[(df[‘IE’]==20.0) & (df[‘CT’]==0.0)]

While loading in the data (which may require a significant amount of memory), the user may also opt to load in only selected columns of data to minimize time and free memory required for the dataset. For instance, the user can only read in the BU, IE, CT columns along with the Cs-137 content in the primary fuel salt and the Xe-135 content in the off-gas tank as follows:

df = pd.read_hdf('path/to/dataset.hdf5’, columns=['BU','CT',‘IE','fuel_Cs137’,‘tank_Xe135’])

## 4 Experimental Design, Materials and Methods

**Fig. 1:**
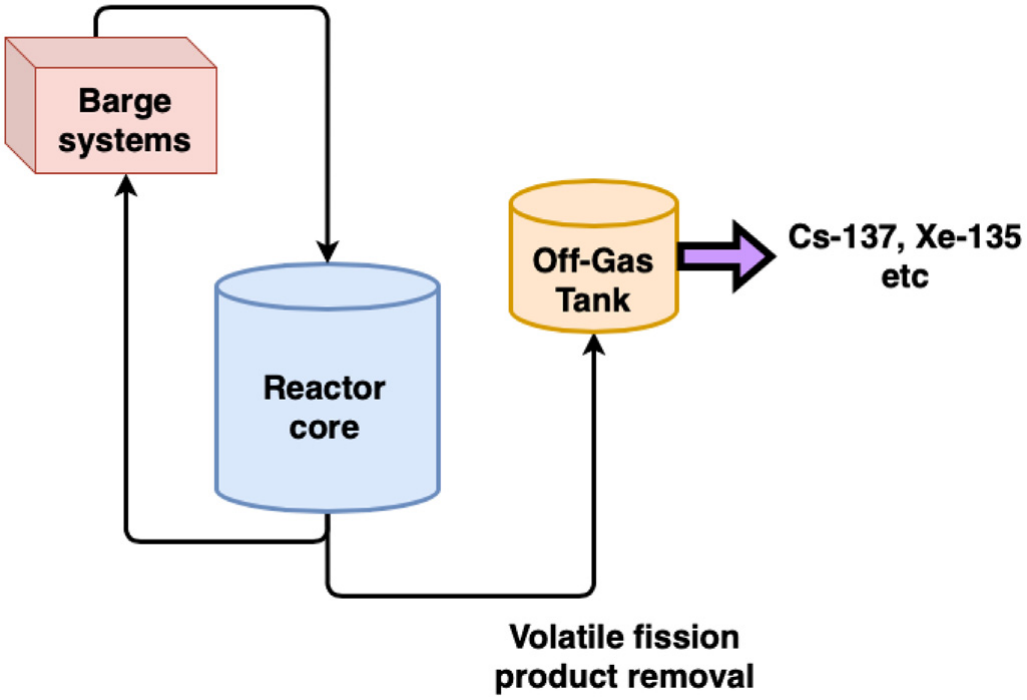
A simplified schematic of the CMSR showing removal of GFPs and VFPs from primary salt to the off-gas tank.

Serpent2 code was used to perform the burnup calculations for the development of the dataset. A 3D model of a concept previously considered by Seaborg Technologies for their floating barge-type reactor was chosen for the development of this dataset. The concept utilizes a FUNaK-type (sodium-potassium fluoride) salt in the primary (with U-235 as driver fuel dissolved in the salt) as well as the secondary circuit (minus the fissile material). Sodium hydroxide (NaOH) was the proposed moderator material for the concept which was envisioned as a 250 MWth/unit reactor. Multiple such units were planned to be placed on a specialized barge, each with an operational lifetime of about 12 years and achieving a terminal burnup of about 20 MWd/kgU. The reactor was to be fueled with HA-LEU (enrichment <20 wt.% U-235) and owing to the highly corrosive nature of both, the salt and the moderator, a specialized proprietary alloy was to be used as structural material. There is no provision for addition of more fissile material during operation, hence the reliance on HA-LEU. The core is designed to accommodate a total of 235 fuel salt channels all of which are surrounded by the moderator. The core also houses control rods (with boron carbide rods) which serve all three functions, start up, shutdown. and power level control. An additional advanced feature present in this CMSR concept is the removal of selected volatile fission products through a designated OGS. This system removes a selection of nuclides from the primary fuel salt and moves them to an off-gas tank. The reactor core parameters that were used in the Serpent2 model for the CMSR concept and the dimensions of the key reactor components are identical to those used in [1]. A simplified schematic of the CMSR is shown in Fig. 1.

**Fig. 2:**
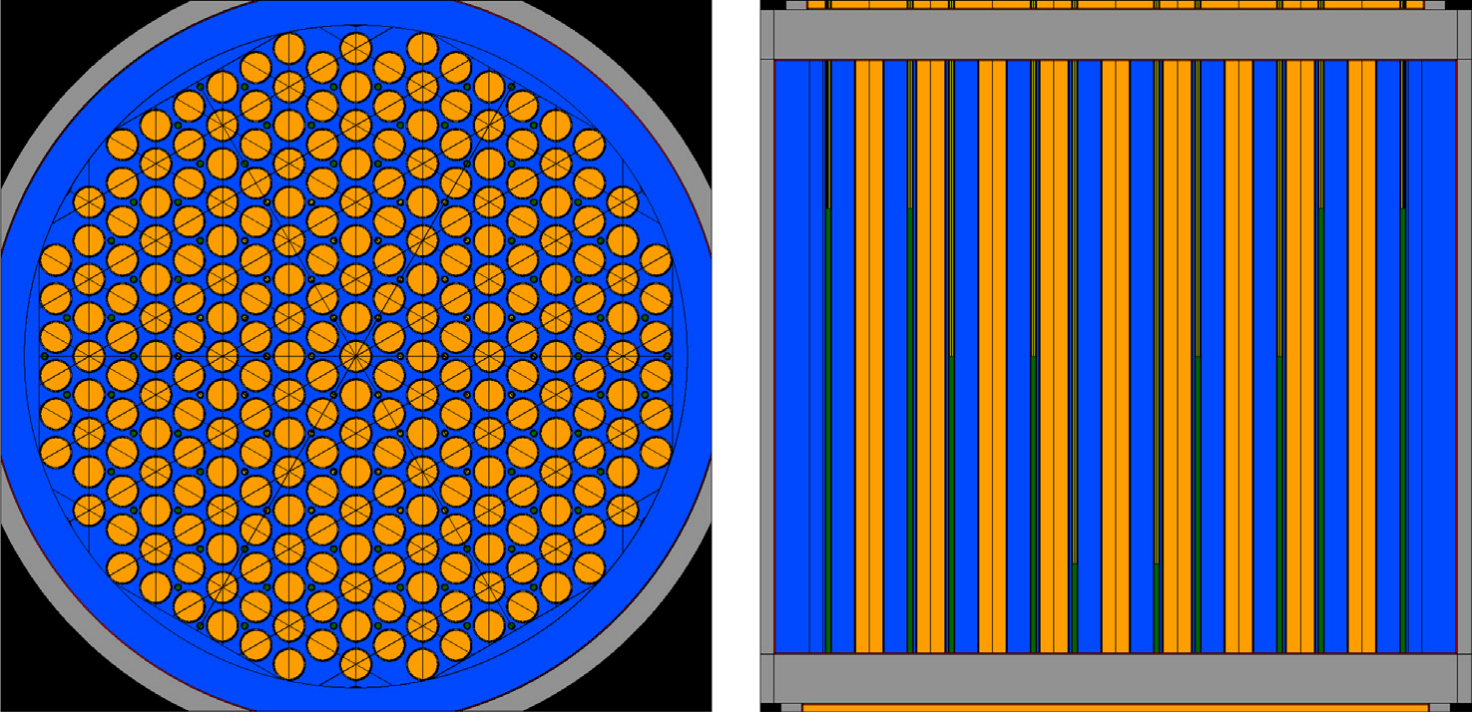
Left: Radial cross-section of the Serpent2 model for the CMSR core showing the hexagonal arrangement of fuel salt channels (orange), structural material in gray, the control rod locations (smaller circles between fuel channels) in green, and moderator (deep blue). Figure also available in [1]. Right: Closer side-view of vertical cross-section of the fuel salt channels and control rod tubes.

The Serpent2 calculations model the irradiation of the primary fuel salt in the core over a pre-selected BIC range (a range that's broad enough to cover the entire operational space of the CMSR). The removal of gaseous and volatile fission products listed in Table 2 was achieved by using the reprocessor feature (*‘rep’*) in Serpent2. The reprocessor feature allows the user to define the rate of removal for a list of nuclides from one region of the geometry to another (more information about this feature available in [16]). In the CMSR model, the reprocessor is used to move the GFPs from the primary salt to another material region (labeled as the off-gas tank). There are several different ”modes” that can be used with the reprocessor that are explained in detail in [3]. For the CMSR dataset, mode 2 was used which allows the user to move material between zones while at the same time, adjust the material amounts and allow a separate depletion calculation (in addition to the depletion of fuel and control material) at each burnup step. Usage of mode 2 in the reprocessor places additional constraints on the mass balance of the overall source system (fuel salt) and fails if the material balance equation computes a negative value after material movement at any burnup step. It should be noted that for the reprocessor feature to function correctly, the off-gas tank need not be part of the geometry in the Serpent2 model. A mere definition of the material in the off-gas tank is sufficient. Further nuances of the reprocessor are explained in greater detail in [[15], [17], [18]]. Fig. 2 shows the Serpent2 model of the CMSR in greater detail.

The JEFF3.3 [19] neutron interaction data, radioactive decay data, fission yields data have been used in the Serpent2 calculations. The moderator has been modeled as a free gas owing to lack of thermal scattering law (TSL) data. Vacuum boundary conditions are applied to the model for the Serpent2 calculations.

**Fig. 3:**
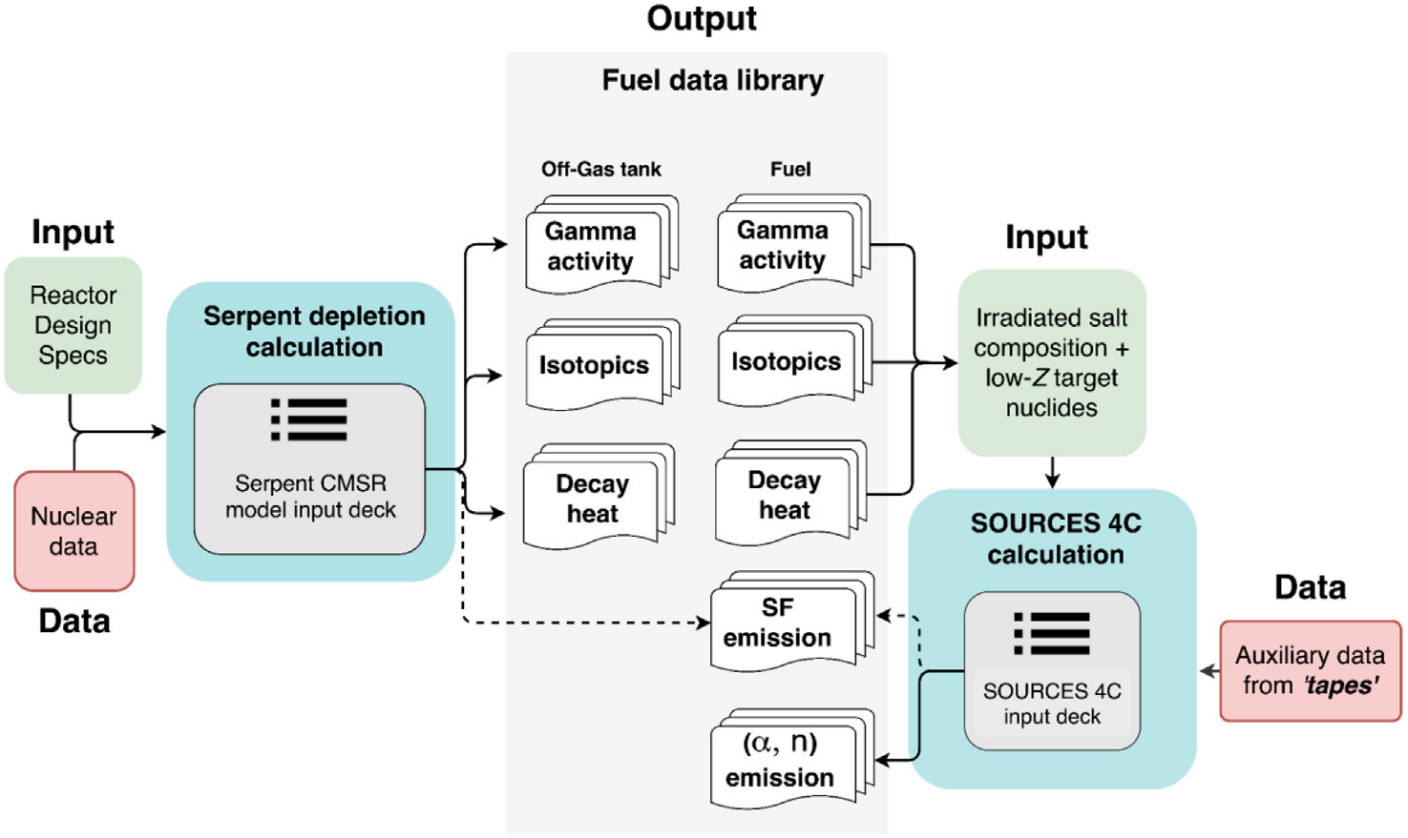
Flow chart showing methodology followed for setting up the fuel library.

As described in the previous sections, total neutron emissions from (ɑ, n) reactions and spontaneous fissions were computed using the SOURCES 4C calculation code. The output files containing depleted material compositions generated using Serpent2 calculations were used to produce input for computing the neutron emission rates. SOURCES code relies on bundled auxiliary data files (called ``tapes”) to obtain nuclear data (such as half-life, branching ratios), cross-section data, neutron yields, material attenuation factors et cetera to run a computation and produces detailed output files with magnitudes and spectra (depending on the user input) for the source-target combination. Here, the source implies the composition of the ɑ-emitting nuclides while target refers to the low-Z nuclides. It is worth noting that the calculation with SOURCES 4C fails if the number of alpha energy groups specified is above 6 when there are more than 10 ɑ-emitters. This, however, was not an issue in the calculation as the dataset includes the total (ɑ, n) emission rate (integrated over all energy bins). The methodology followed for the development of the dataset has been shown in greater detail in Fig. 3.

To summarize, the following are the main assumptions that were used in the modeling and simulations for the creation of this dataset:

1. Serpent 2.1.28 was used in the burnup calculations.
2. JEFF3.3 was used for neutron interaction cross-sections and fission yield data.
3. Within Serpent2, the reprocessor was used in mode 2 to iteratively move material between the core and the off-gas tank at each burnup step.
4. Vacuum boundary conditions were applied to the 3D model of the core.
5. A total of 20 million neutron histories were run to ensure convergence.
6. The neutron emissions from (ɑ, n) and spontaneous fission were computed using the SOURCES 4C code.
7. The data required for the calculations such as cross-sections, stopping powers etc. were supplied as ``tapes” that come bundled with the SOURCES 4C code.
8. 20 alpha energy groups were used for computing the neutron emission rates.

## Limitations

While this dataset is expected to accurately represent the in-core irradiation, composition, and nature of radiation emission from the irradiated salt from the CMSR, if the user of this dataset intends to use it to draw conclusion on reactors of similar design and operational features, they should use due deliberation and carefully examine the full extent of overalls in the reactor types. Additionally, the user is advised that effects arising from Doppler Broadening Rejection Correction (DBRC) have not been factored in the simulations carried out in Serpent2. For further information on the physics and the resulting impact, the user is advised to refer to [20]. Lastly, it must also be mentioned that owing to the large size of the dataset, it is not advisable to load in the entire HDF5 file at once and rather use smaller subsets of the dataset by only loading in the desired columns of data as described earlier in this paper.

## Ethics Statement

(1) This material is the authors' own original work, which has not been previously published elsewhere. (2) The paper is not currently being considered for publication elsewhere. (3) The paper reflects the authors' own research and analysis in a truthful and complete manner. (4) The paper did not involve any human or animal studies and did not involve collection of social media data.

## CRediT authorship contribution statement

**Vaibhav Mishra:** Conceptualization, Methodology, Software, Writing – original draft, Investigation. **Zsolt Elter:** Conceptualization, Methodology, Software, Resources, Writing – original draft, Supervision. **Erik Branger:** Conceptualization, Methodology, Writing – original draft, Supervision. **Sophie Grape:** Conceptualization, Methodology, Writing – original draft, Supervision, Funding acquisition, Project administration. **Sorouche Mirmiran:** Conceptualization, Writing – review & editing.

## Data Availability

Data library of irradiated fuel salt and off-gas tank composition for a molten salt reactor concept produced with Serpent2 and SOURCES 4C codes (Original data) (Mendeley Data).

## Footnotes

1 A total of 42 nuclides are removed from the core at each burnup step at predefined removal rates.
